# A New Beginning

**DOI:** 10.1016/j.shj.2022.100029

**Published:** 2022-03-31

**Authors:** Anthony N. DeMaria

**Affiliations:** Judy and Jack White Chair in Cardiology, Sulpizio Cardiovascular Center, University of California, San Diego, La Jolla, California, USA

This issue represents a major transition for *Structural Heart Journal*, one that in a sense represents a new beginning. We have just completed 5 years of existence, have established our unique role as the only journal devoted to structural cardiovascular disease and the heart team, and have become the official journal of both the Cardiovascular Research Foundation and the Heart Valve Society. With this issue we join hands with a new publisher, Elsevier, who is a giant in the field of medical publishing. This new relationship will offer many opportunities to expand the scope and reach of the journal. In addition, with this issue we enter the rapidly increasing world of open-access publications. Until now the journal has had a sizeable but limited circulation to all registrants of the “Transcatheter” meetings (TCT, TVT, etc) organized by the Cardiovascular Research Foundation. Going forward as an open-access journal, our contents will be available to anyone, anywhere with any interest in structural heart disorders. Of course, as an open-access journal, our financial model will be different, and rather than a subscription fee, authors will be asked to support the cost of publishing their article. We fully recognize the issues involved in making this transition and have taken steps to make it smooth. The publication fees will be held to the lowest level possible, and funds are available to offset the publication fees for authors without financial capability on a priority basis. The transition to open access is in keeping with the accelerating trend in medical publishing to make new information available to as many individuals as soon as possible without the barrier of a financial outlay. It is anticipated that this trend will continue to grow and ultimately become the standard for medical publications. So we welcome everyone to join us in this exciting transition; bookmark the *Structural Heart Journal* webpage, read our original research and review articles, and do submit your interesting material on any aspect of structural heart disease.

## Funding

The authors have no funding to report.

## Disclosure statement

The author reports no conflict of interest.

